# Effect of Nefopam on Dysesthesia, Postoperative Pain, and Satisfaction in Patients with Lumbar Spinal Stenosis Undergoing Spine Surgery: A Double-Blind, Randomized Study

**DOI:** 10.3390/jcm12237468

**Published:** 2023-12-01

**Authors:** Sejong Jin, Yoon Sook Lee, Dahyeon Kim, Bum-Joon Kim, Jae Hwan Kim, Eun-Su Choi

**Affiliations:** 1 Department of Anaesthesiology and Pain Medicine, Korea University Ansan Hospital, Korea University College of Medicine, Ansan-si 15355, Republic of Korea; holicer@korea.ac.kr (S.J.); yslee4719@gmail.com (Y.S.L.); kdh180023@gmail.com (D.K.); anejhkim@korea.ac.kr (J.H.K.); 2 Department of Neuroscience, Korea University College of Medicine, Seoul 02841, Republic of Korea; 3 Department of Neurosurgery, Korea University Ansan Hospital, Korea University College of Medicine, Ansan-si 15355, Republic of Korea; nsbjkim@korea.ac.kr

**Keywords:** dysesthesia, nefopam, postoperative pain, satisfaction, spine surgery

## Abstract

Postoperative residual pain and dysesthesia in patients with lumbar spinal stenosis (LSS) can reduce patient satisfaction. We investigated the effects of nefopam on dysesthesia, postoperative pain, and satisfaction in patients with LSS who underwent spine surgery. A total of 73 patients were randomly assigned to two groups: the nefopam group (*n* = 35), receiving a 20 mL normal saline-based solution containing nefopam 20 mg, and the control group (*n* = 38), which received 20 mL of normal saline 1 h before the end of the operation. Postoperative incisional pain, dysesthesia scores, and overall satisfaction with postoperative pain management were evaluated. The severity of dysesthesia within 12 and 24 h in the nefopam group was significantly lower than that in the control group (2.3 ± 1.9 and 1.7 ± 1.6 vs. 3.3 ± 2.1, and 2.6 ± 1.9, respectively; *p* = 0.029 and *p* = 0.048). Satisfaction scores for postoperative pain management were significantly higher in the nefopam group (3.7 ± 0.6 vs. 3.1 ± 1.0, respectively; *p* = 0.006). The administration of nefopam effectively reduced the severity of dysesthesia within 24 h of surgery in geriatric patients undergoing spine surgery and increased patient satisfaction with postoperative pain management.

## 1. Introduction

Lumbar spinal stenosis (LSS) is a degenerative lumbar disease characterized by compression of the spine’s neural structure through ligamentum flavum hypertrophy, facet joint hypertrophy, disk extrusion, or spondylolisthesis [1,2]. Patients with LSS typically complain of pain and dysesthesia in the lower back, which sometimes involves the lower extremities [1,2]. The importance of LSS is increasing as the population is aging since its prevalence increases with age [3].

A decompressive laminectomy, with or without posterior fusion, is performed when symptoms are uncontrollable with conservative treatment [1,2]. Most patients who undergo surgical intervention report significant symptom relief and high satisfaction; however, approximately 30% complain of residual symptoms after surgery [4,5]. In particular, postoperative residual dysesthesia, such as numbness in the lower extremities, reduces patient satisfaction [6,7]. Surgical site pain and lower back pain are effectively reduced with opioids; however, dysesthesia is less responsive to opioids and takes longer to improve [6,7].

Nefopam is a centrally acting, non-opioid analgesic [8]. Nefopam mediates its analgesic effects through the inhibition of neurotransmitter reuptake and anti-hyperalgesic effects through the modulation of N-methyl-D-aspartate (NMDA) receptors [8]. It also exhibits low-grade anticholinergic activity [8]. Nefopam is an effective and safe analgesic with a strong analgesic effect similar to that of morphine but without respiratory depression [8]. In addition, unlike nonsteroidal anti-inflammatory drugs, nefopam has no side effects such as gastrointestinal bleeding and no antipyretic action that can mask infection-related fever [8].

Several studies investigating various types of surgeries, including abdominal, thoracic, and orthopedic procedures, have documented that nefopam reduces acute postoperative pain and exhibits an opioid-sparing effect [9,10,11,12]. Recently, there has been growing attention paid to the role of nefopam in neuropathic pain [13]. Accumulating preclinical evidence suggests the efficacy of nefopam in neuropathic pain [14,15,16,17,18]. Moreover, Ok et al. reported that nefopam is effective in reducing dysesthesia in endoscopic lumbar discectomy [19].

Insufficient relief of dysesthesia following surgery in patients with LSS can reduce patient satisfaction with the procedure [6,7]. However, there are no studies on the effect of nefopam on postoperative residual pain and dysesthesia in patients with LSS, which can affect patient satisfaction. Based on previous studies [6,7,13,19], intraoperative administration of nefopam is expected to be effective in increasing patient satisfaction by reducing postoperative pain and dysesthesia. Therefore, this study aimed to investigate the effects of nefopam on postoperative pain and satisfaction in patients with LSS undergoing spine surgery.

## 2. Materials and Methods

### 2.1. Study Design and Participants

This study was a prospective, double-blind, randomized controlled trial conducted at the Korea University Ansan Hospital. The study protocol was approved by the Institutional Review Board of Korea University Ansan Hospital (2019AS0165) and registered at the Clinical Research Information Service (https://cris.nih.go.kr, accessed on 7 April 2021, KCT0006067). Written informed consent was obtained from all participants before enrollment.

Eligible participants for this study included patients scheduled for decompressive laminectomy, with or without posterior fusion, with a diagnosis of chronic degenerative LSS with neurogenic claudication. Only adult patients aged 20–75 years with an American Society of Anesthesiologists Physical Status Classification of 1 or 2 were included in the study. The exclusion criteria were as follows: lumbar disc herniation or other spinal disorders, polyneuropathy or peripheral arterial disease, severe pain or disability in other joints (including rheumatoid arthritis or gout), psychiatric disease, sensitivity to test drugs, epilepsy or history of seizure, use of irreversible monoamine oxidase inhibitors, history of myocardial infarction, closed-angle glaucoma, and high risk of urinary retention. All participants were randomly divided into two groups (control and nefopam) in a 1:1 ratio using computer-generated randomization (http://www.randomization.com, accessed on 7 July 2020). An anesthesia nurse who was not involved in this study administered normal saline or nefopam according to the assigned group with the same drug labeling to ensure double blindness.

### 2.2. Intervention and Anesthetic Protocol

Standard monitoring including electrocardiography, non-invasive blood pressure monitor, pulse oximetry, bispectral index (BIS), capnography, and neuromuscular monitoring, and an esophageal stethoscope was applied to the patients throughout the procedure. Total intravenous anesthesia using propofol and remifentanil was performed to induce and maintain general anesthesia. The doses of propofol and remifentanil were adjusted to maintain a BIS between 40–60 and blood pressure and heart rate within 20% of the preoperative values. Rocuronium 0.6 mg/kg was administered initially, and additional rocuronium 0.15 mg/kg was administered when the train-of-four count was 2 or more. Mechanical ventilation was controlled to maintain the end-tidal carbon dioxide partial pressure at 35–40 mmHg with an inhaled oxygen fraction of 40%. Body temperature was monitored using an esophageal stethoscope and maintained above 35 °C with a forced air warmer. Sugammadex (2 mg/kg) was used to reverse muscle relaxation at the end of surgery.

The unlabeled interventional drug solution was administered one hour before the end of surgery. In the nefopam group, a 20 mL solution containing nefopam (Pharmbio Korea Inc, Seoul, Republic of Korea) 20 mg diluted with normal saline was infused over 20 min. In the control group, 20 mL of normal saline was infused over 20 min for the purpose of blinding. At the end of surgery, a patient-controlled analgesia (PCA) device containing a 20 µg/mL fentanyl solution was connected via intravenous access. The PCA device provided a continuous infusion of 10 µg of fentanyl per hour with additional bolus injections of 10 µg of fentanyl every 15 min as needed by the patient. Pethidine (50 mg) was provided as a rescue analgesic upon the patient’s request when the postoperative pain score exceeded 3.

### 2.3. Clinical Assessments and Outcomes

The primary outcome was the severity of dysesthesia in the lower extremities at 12, 24, 48, and 72 h postoperatively, evaluated using an 11-point visual analogue scale ranging from 0 (no discomfort) to 10 points (most extreme discomfort). Dysesthesia was defined as voluntary or involuntary abnormal unpleasant sensations in the lower extremities, including numbness, burning sensation, and tingling.

The secondary outcomes were the severity of postoperative incisional pain in the lumbar area, evaluated using an 11-point visual analogue scale ranging from 0 (no discomfort) to 10 points (most extreme discomfort), incidence of rescue analgesic use, use of PCA, incidence of postoperative nausea and vomiting (PONV), and side effects of nefopam (cardiovascular, digestive, and anticholinergic side effects, including sweating, dry mouth, and tachycardia) at 12, 24, 48, and 72 h postoperatively. Overall satisfaction with postoperative pain management was measured at 72 h postoperatively using the 5-point Likert scale: (1) very unsatisfied, (2) unsatisfied, (3) neutral, (4) satisfied, and (5) very satisfied.

### 2.4. Statistical Analysis

Based on a previous study of leg numbness following lumbar surgery, the severity of leg numbness was 5.9 ± 2.6 preoperatively [6]. Assuming that an improvement of 30% is considered statistically significant, 35 patients were needed for each group with power = 0.8 and α = 0.05. We included 39 patients per group to account for a 10% dropout rate.

Data are expressed as means ± standard deviation or numbers (percent). The Chi-squared test was performed for comparing categorical data. Continuous variables were compared using the independent *t*-test. All statistical analyses were performed using SPSS Statistics ver. 21.0 (IBM SPSS, Chicago, IL, USA). A *p*-value of <0.05 was considered statistically significant.

## 3. Results

**Figure 1:**
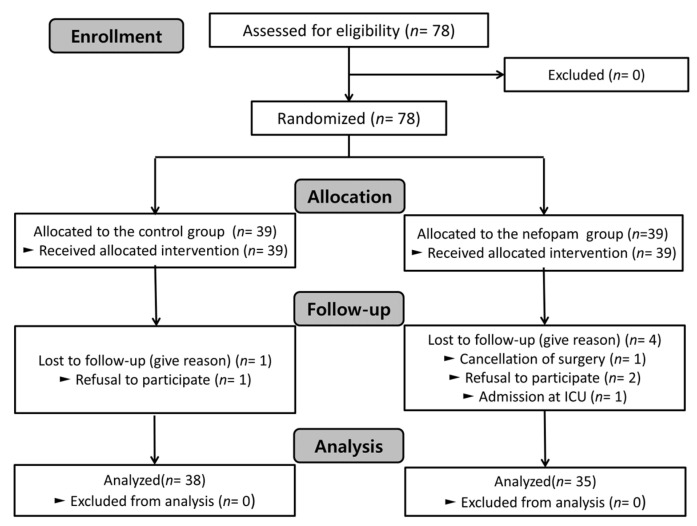
Consort flow diagram.

A total of 78 patients were enrolled and randomly allocated to two groups. One patient in the control group (refusal to participate) and four patients in the nefopam group (one due to cancellation of surgery, two due to refusal to participate, and one due to ICU admission) were excluded. Therefore, 38 and 35 patients in the control and nefopam groups, respectively, were analyzed (Figure 1).

Demographic and operative data are presented in Table 1. There was no statistical difference in demographic data between the patients in both groups. The severity of spinal stenosis, evaluated via the Lee grading system using magnetic resonance imaging [20], showed no significant difference between the control and nefopam groups. Operative data did not significantly differ between the two groups. In addition, parameters, including intraoperative blood loss and operation time, were consistent with those reported in a previous study [21].

The scores for dysesthesia in the lower extremities, preoperative back pain, and postoperative incisional pain in the lumbar area are shown in Table 2. The preoperative severities of leg dysesthesia and back pain did not differ between the control and nefopam groups. In both groups, the severity scores for the lower extremities and postoperative pain in the lumbar area decreased over time. In particular, the dysesthesia score in the lower extremities of the nefopam group was significantly lower than that of the control group at 12 and 24 h postoperatively. In contrast, the dysesthesia scores in the lower extremities at 48 and 72 h postoperatively were comparable between the two groups. The postoperative incisional pain scores were lower in the nefopam group than in the control group at 12, 24 and 48 h postoperatively, although the difference was not statistically significant. Notably, the postoperative incisional pain scores at 72 h did not differ between the control and nefopam groups.

There was no difference in the amount of PCA (fentanyl-based) or additional use of rescue analgesics (pethidine) between the two groups (Table 3). After 24 h, one patient in the control group discontinued PCA because of nausea and vomiting, and after 48 h, another patient in the control group discontinued PCA because of the same reasons. Patient satisfaction scores for postoperative pain management were significantly higher in the nefopam group than in the control group (Table 3).

There were no significant differences in the incidence of PONV or nefopam side effects between the two groups (Table 4).

## 4. Discussion

The results of this study indicate that, in geriatric patients who underwent spine surgery, dysesthesia in the lower extremities was reduced for 24 h post-operation without any additional severe side effects following the intraoperative administration of nefopam (20 mg) before the end of surgery. In addition, nefopam (20 mg) reduced postoperative pain for 48 h after surgery, although this was not statistically significant. Additionally, nefopam (20 mg) administration increased patient satisfaction with postoperative pain control, without any side effects.

Nefopam, a non-opioid analgesic derived from the non-sedative benzoxazocine, acts centrally to alleviate pain through an unconventional mechanism. Operating on the brain and spinal cord, its primary analgesic mechanisms involve elevating serotonin, norepinephrine, and dopamine activity [22,23], and inhibiting glutamate secretion by modulating calcium and sodium channels [24,25,26,27]. According to previous studies, nefopam not only affects postoperative pain [28,29], but is also effective for neuropathic pain [13,19]. This efficacy in neuropathic pain is attributed to its antidepressant and anticonvulsant properties, achieved through the antagonism of NMDA glutamate receptors, sympathetic blockade, the inhibition of calcium influx and intracellular cGMP formation, and the prevention of the activation of voltage-sensitive calcium channels followed by NMDA receptor-dependent neurotoxicity [27,30].

Previous studies investigating the effectiveness of nefopam in patients undergoing spine surgery have yielded mixed results. Some studies have reported that nefopam did not reduce PCA or opioid consumption during the first 3 d post-operation [31,32,33]. In contrast, another study found that the group treated with nefopam experienced less pain during ambulation and had a shorter hospitalization period than the control group [12]. In our study, the nefopam group reported reduced postoperative pain; however, the difference was not statistically significant. Moreover, there was no significant reduction in the use of fentanyl-based PCA. This discrepancy might be partly attributed to the relatively low single dose of nefopam (20 mg) used in our study, considering that the median effective dose in moderately invasive surgery is 28 mg [34].

Conversely, nefopam demonstrated a significant effect in reducing postoperative dysesthesia for up to 1 d, unlike postoperative pain. As postoperative dysesthesia is considered a part of neuropathic pain [35], this result suggests that nefopam might have an effect on neuropathic pain, which is consistent with previous studies [13,19]. Previous studies have suggested that the mechanism of catheter-related bladder discomfort [36] and chronic pain after breast cancer surgery [37] involves muscarinic receptor stimulation and the release of neurotransmitters such as serotonin, dopamine, and norepinephrine. These studies demonstrated the effects of nefopam for up to 24 h, which is consistent with our findings. This suggests that nefopam may be effective in managing postoperative dysesthesia in patients with LSS undergoing spine surgery, given its similar mechanism of action.

In our study, a single bolus of 20 mg nefopam was administered immediately after surgery. Previous studies have indicated that the median effective dose of nefopam in minor-to-moderate surgeries ranges from 18 to 28 mg [34,38,39]. The effects of a single bolus of 20 mg nefopam have been explored under various conditions. It has demonstrated efficacy for up to 72 h in catheter-related bladder discomfort [36] and for up to three months in chronic pain after breast cancer surgery [37]. As the majority of our study participants were older patients, 20 mg of nefopam was selected to ensure safety in light of the age-related decline in liver and kidney function. Additionally, since a previous study comparing pre- and post- operative administration of nefopam found no significant difference [33], the timing of administration was unlikely to have influenced the results. Nefopam was administered postoperatively in our study.

According to a previous study on the clinical course of pain and disability following surgery for spinal stenosis, most patients experienced a 50% reduction in pain three months after surgery; however, mild-to-moderate pain and disability persisted even after 60 months [6,40]. In particular, dysesthesia, such as leg numbness, showed a slower improvement than leg pain [6]. Factors that affect patient satisfaction after spine surgery include surgical outcomes, meeting patient expectations, and an improvement in pain and disability [41,42,43,44,45,46]. In particular, in older patients, preoperative expectations are highly correlated with postoperative satisfaction; therefore, narrowing the gap between expectations and satisfaction is important to increase satisfaction [46]. Therefore, reducing the patient’s pain or dysesthesia may help in increasing the satisfaction associated with surgery and postoperative pain management. In this study, the most important symptom experienced by patients was dysesthesia, and this symptom was significantly reduced 24 h after surgery, which affected satisfaction with postoperative pain control and created a significant difference between two groups.

This study had several limitations. First, patients were observed for only 3 d, providing short-term information. Previous studies have suggested that nefopam may have beneficial effects on chronic pain even after three months [37]. Notably, most patients who undergo spine surgery take six months or longer to experience a 50% improvement in their symptoms [6]. Therefore, a long-term follow-up is essential to fully understand nefopam’s effectiveness. Second, the absence of an exclusive nefopam-only PCA group makes it challenging to directly compare our results with those of previous studies. If such a group had been included, it might have offered clearer evidence of nefopam’s effects on dysesthesia. Third, despite achieving statistical significance, the clinical relevance of our findings appears to be modest, reflecting only a one-point difference in leg dysesthesia score at 12 h post-surgery (3.3 versus 2.3). However, in light of the increasing emphasis on opioid-sparing analgesia, the amelioration of pain through the use of non-opioid medications could bear clinical importance. In addition, the relatively low dose of nefopam administered in this study is a noteworthy factor to explain its limited impact [26]. Fourth, our study had a relatively small sample size, which was determined through statistical estimation based on previous studies. This may have introduced bias. Additionally, it should be acknowledged that nefopam is a drug not yet approved in several countries, including the United States. Finally, the inclusion of many older patients may have affected the accuracy of patient responses. Although the study initially included adults aged ≥ 20 years, in practice, the patient population skewed towards an older population, with the average age corresponding to the older category, due to the epidemiological characteristics of patients with LSS. Older patients may find it difficult to provide precise responses, particularly when distinguishing between back pain and leg dysesthesia. However, considering that the patients in our study reported more dysesthesia in their legs than back pain preoperatively, it may have been easier for them to differentiate leg dysesthesia from postoperative incision pain. This may aid in assessing the differential effectiveness of nefopam for pain.

This study highlights the potential pivotal role of nefopam in postoperative pain management, specifically in the alleviation of postoperative neuropathic pain. These findings hold significant value, as they contribute to the development of a suitable PCA regimen that aligns with patient expectations. This alignment is a crucial factor in enhancing postoperative satisfaction among patients while minimizing the risk of severe side effects. Further research and long-term follow-up studies are warranted to validate these findings and provide a more comprehensive understanding of nefopam’s role in postoperative pain management.

## 5. Conclusions

In this study, the nefopam-treated group exhibited a reduction of up to 30% in dysesthesia in the lower extremities compared to the control group for up to 24 h postoperatively. Additionally, they reported a 16% higher satisfaction with overall pain control until 72 h postoperative. However, there were no significant differences observed in incisional pain or the usage of analgesics. Consequently, the administration of nefopam appears to be effective in reducing postoperative dysesthesia and satisfaction in patients with LSS undergoing spine surgery.

## Figures and Tables

**Table 1:** Demographic and operation data.

	Control Group (*n* = 38)	Nefopam Group (*n* = 35)	*p* Value
Demographic data			
Age (year)	66.7 ± 7.8	63.9 ± 6.7	0.101
Body-mass index	25.3 ± 3.0	25.9 ± 4.4	0.505
ASA (1/2)	2/36	5/30	0.276
Sex (female/male)	23/15	24/11	0.473
Past history			
Hypertension	23 (60.5%)	17 (48.6%)	0.305
Diabetes mellitus	7 (18.4%)	9 (25.7%)	0.452
Respiratory disease	6 (15.8%)	7 (20.0%)	0.639
Neuromuscular disease	7 (18.4%)	6 (17.1%)	0.887
Endocrinological disease	8 (21.1%)	3 (8.6%)	0.136
Severity of spinal stenosis ^1^			
Grade 0 (normal)	0 (0%)	0 (0%)	0.887
Grade 1 (mild)	0 (0%)	0 (0%)	
Grade 2 (moderate)	7 (18.4%)	6 (17.1%)	
Grade 3 (severe)	31 (81.6%)	29 (82.9%)	
Operation data			
Number of spine level	1.7 ± 0.9	1.9 ± 1.0	0.484
Estimated blood loss (mL)	360.5 ± 257.1	331.4 ± 243.2	0.622
Crystalloid (mL)	1030.9 ± 955.5	1015.7 ± 872.8	0.944
Blood Transfusion	1 (2.6%)	3 (8.6%)	0.265
Operation time (min)	178.9 ± 116.6	171.5 ± 94.0	0.768
Anesthetic time (min)	226.4 ± 123.8	223.3 ± 98.4	0.908

The values are presented as number (%) or mean ± standard deviation. ^1^ The Lee grading system using magnetic resonance imaging. Abbreviations: ASA; American Society of Anesthesiologists Physical Status Classification.

**Table 2:** The scores for dysesthesia in the lower extremities, preoperative back pain, and postoperative incisional pain.

	Control Group (*n* = 38)	Nefopam Group (*n* = 35)	*p* Value
Preoperative leg dysesthesia	6.2 ± 2.8	6.3 ± 2.5	0.907
Postoperative leg dysesthesia			
12 h	3.3 ± 2.1	2.3 ± 1.9	0.029 *
24 h	2.6 ± 1.9	1.7 ± 1.6	0.048 *
48 h	1.9 ± 1.8	1.7 ± 1.5	0.695
72 h	1.7 ±1.8	1.7 ± 1.7	0.988
Preoperative back pain	4.1 ± 2.7	4.4 ± 3.5	0.686
Postoperative incisional pain			
12 h	4.1 ± 3.1	3.5 ± 2.4	0.396
24 h	3.5 ± 2.5	3.3 ± 1.7	0.717
48 h	3.1 ± 1.9	2.7 ± 1.9	0.449
72 h	2.3 ± 1.5	2.4 ± 1.9	0.788

Values are presented as mean ± standard deviation. * *p* < 0.05.

**Table 3:** Usage of patient-controlled analgesia (fentanyl-based) and rescue analgesics (pethidine) and the score of patient satisfaction for pain control.

	Control Group (*n* = 38)	Nefopam Group (*n* = 35)	*p* Value
PCA (mL)			
12 h	14.7 ± 7.5	16.3 ± 5.5	0.297
24 h	14.1 ± 7.5	15.7 ± 4.9	0.262
48 h	13.6 ± 6.1	14.8 ± 7.6	0.497
72 h	9.3 ± 4.8	11.6 ± 6.4	0.132
Usage of rescue analgesics (one time/two times)			
12 h	6 (15.8%)/7 (18.4%)	8 (22.9%)/6 (17.1%)	0.845
24 h	1 (2.6%)/4 (10.5%)	1 (2.6%)/4 (11.4%)	0.990
48 h	1 (2.6%)/2 (5.3%)	2 (5.7%)/0 (0.0%)	0.321
72 h	0 (0.0%)/2 (5.3%)	2 (5.7%)/1 (2.6%)	0.294
Satisfaction score	3.1 ± 1.0	3.7 ± 0.6	0.006 *

Values are presented as number (%) or mean ± standard deviation. Abbreviations: PCA; patient-controlled analgesia. * *p* < 0.05.

**Table 4:** Incidence of postoperative nausea and vomiting and side effects of nefopam.

	Control Group (*n* = 38)	Nefopam Group (*n* = 35)	*p* Value
Postoperative nausea and vomiting			
12 h	6 (15.8%)	5 (14.3%)	0.858
24 h	9 (23.7%)	5 (14.3%)	0.308
48 h	6 (15.8%)	5 (14.3%)	0.858
72 h	4 (10.5%)	2 (5.7%)	0.455
Complications of nefopam (1/2/3/4)			
12 h	2/1/0/0	0/0/2/0	0.176
24 h	2/1/1/0	3/1/2/0	0.847
48 h	3/2/0/2	1/0/2/0	0.141
72 h	1/1/0/0	1/0/2/0	0.374

The values are presented as number (%). Complications 1–4 are dizziness, dry mouth, drowsiness, and constipation, respectively.

## Data Availability

The data supporting the findings of this study are available from the corresponding author upon reasonable request.
